# Supplementary material for: Comparative multiomics analysis of cell physiological state after culture in a basket bioreactor
Source: Sci Rep. 2022 Nov 23;12:20161. doi: 10.1038/s41598-022-24687-4 (PMC9686226; doi:10.1038/s41598-022-24687-4)
Supplement: Supplementary file 1 — Supplementary Information 1. [file 41598_2022_24687_MOESM1_ESM.zip › raw data/Transcriptomic raw data/6.Enrichment/2.KEGG/TE1vsTC1/DOWN/TE1vsTC1.down_KEGGbar.pdf]

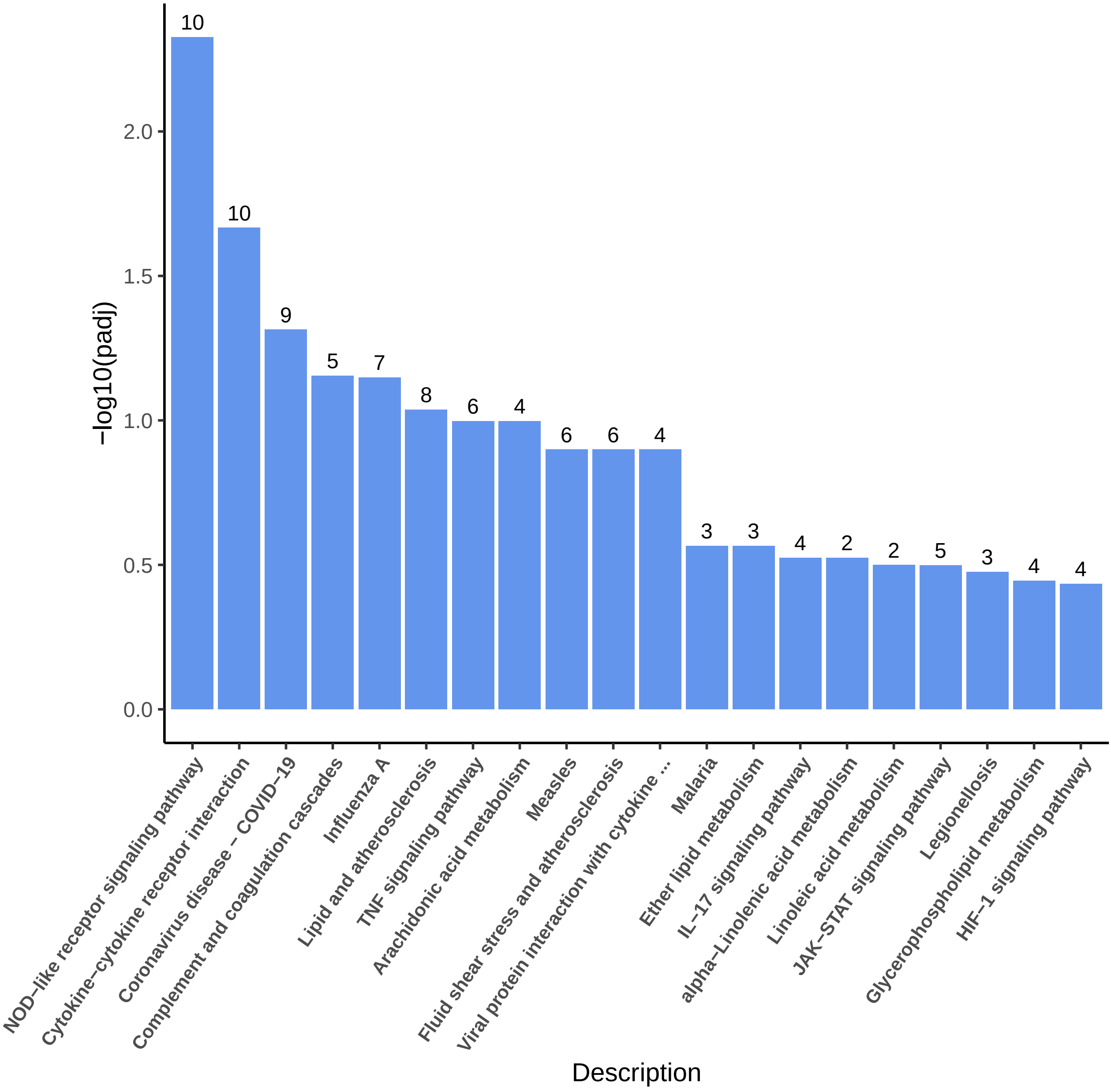

−log10(padj)
Description
0.0
0.5
1.0
1.5
2.0
10
10
9
5
7
8
6
4
6
6
4
3
3
4
2
2
5
3
4
4
NOD−like receptor signaling pathway
Cytokine−cytokine receptor interaction
Coronavirus disease − COVID−19
Complement and coagulation cascades
Influenza A
Lipid and atherosclerosis
TNF signaling pathway
Arachidonic acid metabolism
Measles
Fluid shear stress and atherosclerosis
Viral protein interaction with cytokine ...
Malaria
Ether lipid metabolism
IL−17 signaling pathway
alpha−Linolenic acid metabolism
Linoleic acid metabolism
JAK−STAT signaling pathway
Legionellosis
Glycerophospholipid metabolism
HIF−1 signaling pathway
